# Missing trial results: analysis of the current publication rate of studies in pediatric dialysis from 2003 to 2020

**DOI:** 10.1007/s00467-022-05553-x

**Published:** 2022-04-23

**Authors:** Christian Patry, Alexander Fichtner, Britta Höcker, Markus Ries, Claus Peter Schmitt, Burkhard Tönshoff

**Affiliations:** Department of Pediatrics I, University Children’s Hospital Heidelberg, Heidelberg, Germany

**Keywords:** Pediatric dialysis, Publication rate, Time to publication, FDA Amendments Act

## Abstract

**Background:**

Decision-making in the field of pediatric dialysis requires evidence from clinical trials, but, similar to other fields of pediatric medicine, might be affected by a low trial publication rate.

**Methods:**

We analyzed the current publication rate, the time to publication, and factors that might be associated with both rate of and time to publication in pediatric dialysis studies registered as completed on ClinicalTrials.gov from 2003 until November 2020.

**Results:**

Fifty-three respective studies were identified. These enrolled 7287 patients in total. 28 of 53 studies (52.8%) had results available. We identified a median time to publication of 20.5 months (range, 3–67). Studies published after the FDA Amendments Act establishment in 2007 were published faster (P = 0.025). There was no trend toward a higher publication rate of studies completed more recently (P = 0.431). 26 of 53 studies (49.1%) focused on medication and control of secondary complications of kidney failure. 12 of 53 studies (22.6%) enrolled only children, were published faster (P = 0.029) and had a higher 5-year publication rate (P = 0.038) than studies enrolling both children and adults. 25 of 53 studies (47.1%) were co-funded by industry. These were published faster (P = 0.025).

**Conclusions:**

Currently, only 52.8% of all investigated studies in pediatric dialysis have available results, and the overall median time to publication did not meet FDA requirements. This might introduce a publication bias into the field, and it might negatively impact clinical decision-making in this critical subspecialty of pediatric medicine.

**Graphical abstract:**

A higher resolution version of the Graphical abstract is available as Supplementary information

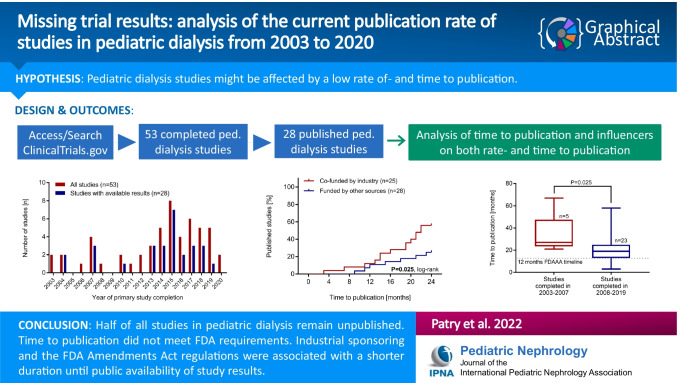

**Supplementary Information:**

The online version contains supplementary material including a graphical abstract available at 10.1007/s00467-022-05553-x.

## Introduction

Chronic kidney disease in childhood is associated with a significant morbidity and mortality [1]. Current data from the United States Renal Data System (USRDS) registry show that, depending on patient age at reaching chronic kidney disease stage 5, more than 60 to 90% of all affected children do not receive a preemptive kidney transplantation and require chronic dialysis treatment [2]. Yet, even on dialysis, problems related to kidney failure persist and complications related to the chosen dialysis mode itself reduce long-term survival of the affected children [3–7].

Improvement of long-term outcome of children on dialysis depends largely on evidence derived from pediatric dialysis-specific clinical studies with a focus on patient management, dialysis technique and efficiency. However, currently by far not all completed studies in pediatric medicine get published. Recent literature shows that the publication rate in pediatric clinical research ranges between only 60 and 70% [8–11]. Such an incomplete publication rate, if caused by selective non-publication [12], might give rise to a publication bias. This is a common concern in evidence-based medicine, because it might negatively impact clinical decision-making and thereby negatively affect patient outcome [13]. In addition to the AllTrials Campaign and the International Committee of Medical Journal Editors (ICMJE)-statement from 2004, which both require that all clinical trials shall be registered, the FDA Amendments Act (FDAAA) stated for the first time in 2007 that all clinical trials should not only be registered but also have available results published within 12 months after primary completion [14–16]. This act has currently been enforced as US federal law in January 18, 2017 [17]. Despite these measures, the problem of non-publication and untimely publication of medical studies still remains, as has been shown recently [18].

We hypothesize that the specialized field of pediatric dialysis might also be affected by a publication bias. However, research on this specific question has not been performed yet. Thus, as a first measure, we designed this study to investigate the proportion of completed studies with unavailable results in the field of pediatric dialysis. Furthermore, we investigated the respective duration from primary study completion until publication and analyzed factors with a possible impact on time to publication or on the publication rate.

## Materials and methods

This is an observational study on completed clinical studies in pediatric dialysis.

### ClinicalTrials.gov database query

**Fig. 1:**
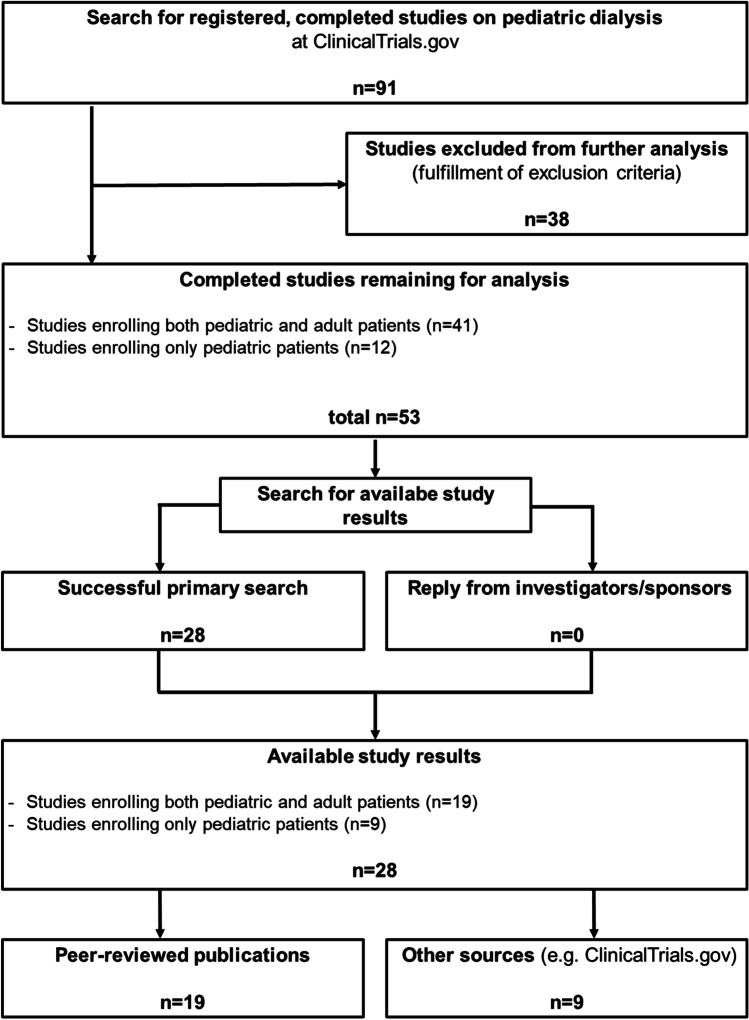
Study flow diagram. Identification of studies with and without available study results on pediatric dialysis registered as completed on ClinicalTrials.gov until November 20, 2020. Primary search terms were: “dialysis”, “hemodialysis”, “hemofiltration”, “hemodiafiltration” and “peritoneal dialysis”. ClinicalTrials.gov search specifications were “child” and “completed”

We designed and performed this analysis in adherence to the STROBE criteria [19]. First, we assessed ClinicalTrials.gov on November 20, 2021 and searched for all registered and completed clinical studies on pediatric dialysis starting from the date of the first primary study completion (October 15, 2003) until November 20, 2020. We used the primary search terms “Dialysis”, “Hemodialysis”, “Hemofiltration”, “Hemodiafiltration” and “Peritoneal dialysis”. Each search was specified with the selection parameters “child” and “completed”. ClinicalTrials.gov defines the selection parameter “child” as an age range from birth until 17.9 years. After that, we checked the raw data generated by ClinicalTrials.gov for plausibility and duplicates. We excluded: (i) studies which did not primarily address dialysis, (ii) studies which did not include children, as assessed in respective publications, and (iii) studies which had no specified completion date given. Figure 1 shows the methodological flowchart of our analysis.

### Items of analysis

We analyzed the following categorical or continuous variables: (i) availability of study results, (ii) patient age as written in the study description on ClinicalTrials.gov or if available as written in the respective available study results, (iii) duration from primary study completion (defined by the US federal regulations as “the date that the final study participant was examined or received an intervention for the purpose of the final collection of data for the primary outcome” [20]) until availability of study results, (iv) number of enrolled patients in each study, (v) design, and (vi) type of sponsor. We further categorized the studies into two major research topics as appropriate: (i) pediatric dialysis studies with primary focus on technical aspects and efficiency of dialysis procedure as well as prescription; (ii) pediatric dialysis studies with a primary focus on medication and control of secondary complications of kidney failure. Studies that could not be assigned to one of the two categories were grouped together under the category "others”. We defined a pediatric patient as a patient with an age below 18 years. We further subdivided studies into those which only enrolled pediatric patients, named “pediatric studies”, and those which enrolled both pediatric and adult patients, named “combined adult and pediatric studies”. The date when the FDAAA became effective (September 27, 2007) and the primary completion date of registered studies as defined by the FDAAA (see above) were used as reference points.

## Analysis of available study results and time to publication

In accordance with the requirements stated in the FDAAA, a study in our analysis was defined as published if we found a peer-reviewed publication primarily via Pubmed or if we found other sources of available study results. In the latter case, these were study results posted on ClinicalTrials.gov in most cases. The results of one study were directly posted by the industrial sponsor for public availability in a product description sheet. If we did not find available results of a study by the means described above, we directly contacted principal investigators or sponsors if named by ClinicalTrials.gov regarding data requests; hereby, we did not receive any answer. The investigators of five studies could not be contacted because of missing contact information. The closure of database was the end of our search for available study results on November 20, 2021. We identified the latest impact factors of the respective journals of peer-reviewed publications by accessing the respective journal homepages. We analyzed the time from primary completion until the earliest date of availability of study results. In this time-to-publication analysis (in accordance with the FDA regulations), we considered the date of first posting of results on ClinicalTrials.gov, even if there was a possible peer-reviewed publication later. This was the case regarding four studies. The FDAAA in principle only applies to so called “applicable clinical trials” [20], which we could not clearly identify as such based on the information given on ClinicalTrials.gov. However, as we felt that a timely publication of data should be the general goal of all clinical trials involving human subjects, we evaluated the time to publication referring to the timeline of 12 months as mandated by the FDA for all studies in our analysis. We specifically report the proportion of peer-reviewed publications, as we considered especially those to be scientifically valid. In addition to the proportion of currently available study results, we analyzed the percentage of publications within 12 months and the rate of publications within 24 months (“2-year publication rate”) and 60 months (“5-year publication rate”) after primary study completion.

### Statistical analysis

Standard methods of descriptive statistics were applied, missing data were not imputed. We reported data as median (interquartile range, IQR) and range, when there was non-Gaussian distribution of values. The time from primary completion until availability of study results was set to 0 months if results were available before the primary completion date. Parametric and non-parametric tests were performed as appropriate and are indicated in the tables and figures in the results section. We performed Cox regression analyses based on the Kaplan–Meier survival method to analyze differences regarding the 2-year publication rate and the 5-year publication rate. A *P* value < 0.05 was considered as statistically significant for all tests. All calculations were performed using GraphPad Prism v.7.01 (GraphPad Software, Inc., California, USA) and IMB SPSS Statistics v.26.0.0.0 (IBM Corp ©). Figures were created with the Software GraphPad Prism v.7.01.

## Results

### Overview of studies, participants, funding, design, and currently available results

Our analysis included 53 completed studies on pediatric dialysis with a cumulative enrollment of 7287 study participants (Table 1). 52.8% of these studies were published at time of database closure, comprising 4025 enrolled participants. Hence, study results from 3262 participants are currently missing. Supplementary Table 1 gives detailed information on each study stratified according to research topic, primary completion date, design, phase, funding and patient population (pediatric study or combined adult and pediatric study). One study only included female patients, one study did not specify patient sex. 22.6% of all studies enrolled only pediatric patients, and 75% of those were published already. Pediatric studies enrolled a cumulative number of 616 patients (Table 1, Fig. 1), with a significantly lower median enrollment of 17 patients (IQR 11.3–62; range 3–321) compared to 50 patients (IQR 24–147; range 7–1892) in studies including both children and adult patients (P = 0.016). Nearly half of all studies were co-funded by industry and one-fourth of all studies had a randomized controlled trial (RCT) design. 60% of industry-funded studies and 64.3% of all RCTs had currently available study results (Table 1). There were three phase I trials, five phase II trials, six phase III trials and six phase IV trials. The median number of patients was 46.0 (IQR 19.5–121.5; range 3–1892) per study in the overall analysis (n = 53), 26 (IQR, 16.5–94.8; range 6–1892) per study with available results (n = 28), 17 (IQR 11.3–62.0; range 3–321) per pediatric study, 57 (IQR 12–204; range 3–1892) per study co-funded by industry and 24 (IQR 12–58.5; range 6–632) per RCT. The results of 19 studies had been published after peer-review. Table 1 shows peer-reviewed publications stratified by research topics, study design and type of funding. The results of seven studies were only posted to ClinicalTrials.gov, two studies had results available which could be found in other sources (product sheet, not peer-reviewed journal with free access via the internet). The median impact factor of peer-reviewed published studies was 3.17 (Table 2). Fourteen of 19 (73.7%) studies with peer-reviewed publication showed at least one statistically significant result compared to none of the studies which only had results posted on ClinicalTrials.gov.

**Table 1:** Number of studies, publication rate and number of patients in completed studies on pediatric dialysis registered at ClinicalTrials.gov

Characteristic	Number of studies	Results available (% of number of studies)	Peer-reviewed results (% of number of studies)	Patient number enrolled	Patient number enrolled in studies with available results (% of patient number enrolled)
All studies	53	28 (52.8)	19 (35.8)	7287	4025 (55.2)
Pediatric studies, n (% of 53)	12 (22.6)	9 (75.0)	6 (50.0)	616	542 (87.9)
Research topics (% of 53)					
Medication and control of secondary complications	26 (49.1)	13 (50.0)	7 (26.9)	2869	1324 (46.1)
Dialysis technique and dialysis efficiency	20 (37.7)	11 (55.0)	8 (40.0)	1965	639 (32.5)
Others	7 (13.2)	4 (57.1)	4 (57.1)	2453	2062 (84.1)
Study design, n (% of 53)					
Randomized controlled trials	14 (26.4)	9 (64.3)	8 (57.1)	621	398 (64.1)
Non-randomized interventional trials	18 (33.9)	12 (66.7)	7 (38.9)	1376	1155 (83.9)
Observational studies	21 (39.6)	7 (33.3)	4 (19.0)	5290	2472 (46.7)
Type of funding, n (% of 53)					
Co-funded by industry	25 (47.1)	15 (60.0)	9 (36.0)	4725	3474 (73.5)
Funded by academia	28 (52.8)	13 (46.4)	10 (35.7)	2562	551 (21.5)

**Table 2:** Impact factors of journals of peer-reviewed, published studies in pediatric dialysis stratified according to the time from primary completion till availability of study results

Studies with peer-reviewed publication	n	Impact factor	P value
		median (IQR)	
Published studies	19	3.71 (3.71–8.23)	-
Studies published early^a^	16	3.71 (2.71–7.59)	0.693^c^
Studies published late^b^	7	3.71 (3.71–6.06)	

^a^Early publication is defined as a publication within 24 months after primary completion of the respective study;

^b^Late publication is defined as a publication later than 24 months, but not later than 60 months after primary completion of the respective study;

^c^Chi-square test. 1 study was published later than 60 months after completion

### Research topics

Table 1 shows completed studies in pediatric dialysis stratified by research topic. 26 of 53 studies (49.1%) focused primarily on medication and control of secondary complications of kidney failure, half of those studies had results available at the time of database closure, and 6 studies had a randomized design. 20 of 53 studies (37.7%) focused on dialysis technique and efficiency. Here, 55% of those have already been published and 7 studies had a randomized design (Table 1). 20 of 26 studies (76.9%) on medication and control of secondary complications were co-funded by industry. The cumulative number of enrolled patients (n = 2869 participants) was highest in studies with focus on medication and control of secondary complications. Unpublished studies also addressed relevant clinical research questions in pediatric dialysis (Table 1).

### Number of studies and participants by year of study completion

**Fig. 2:**
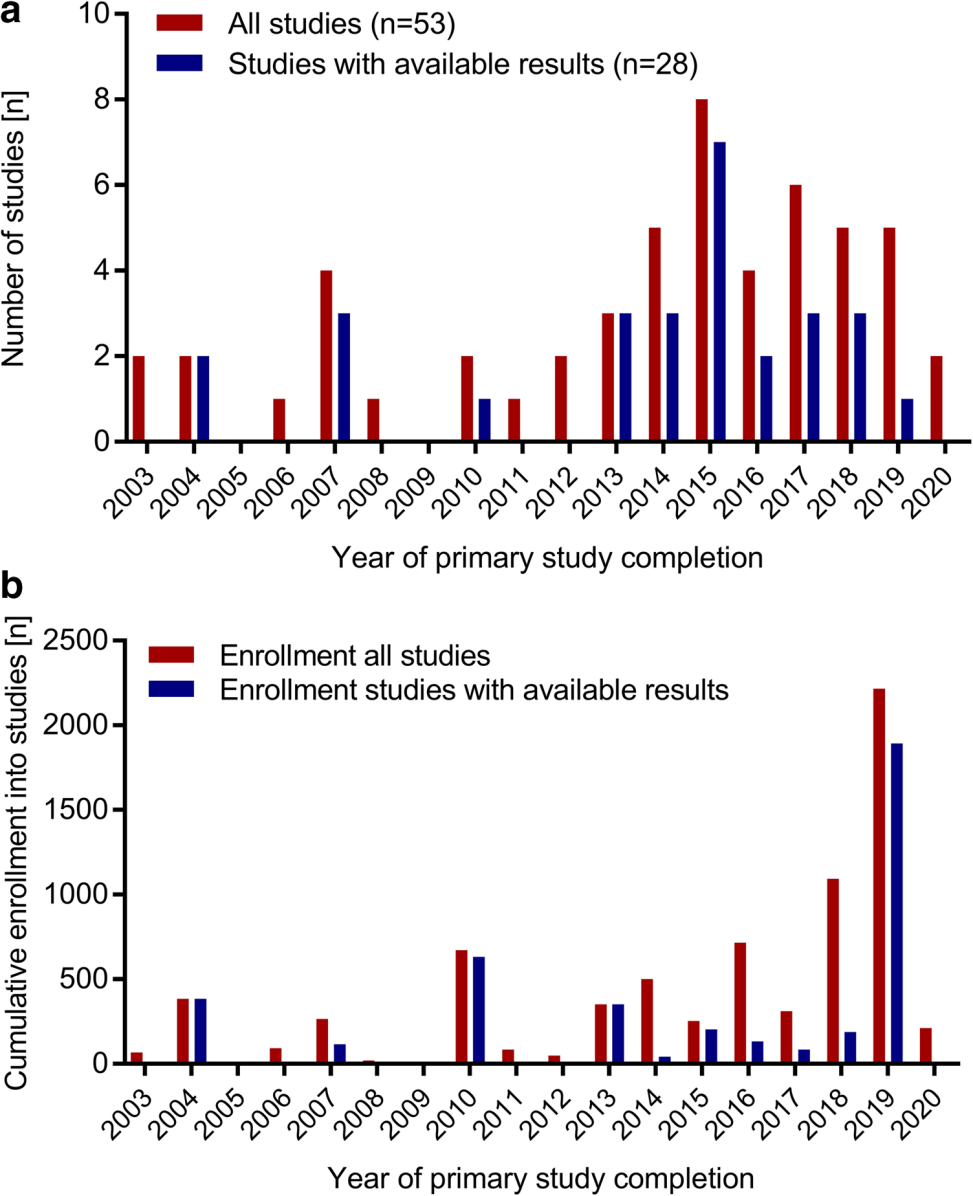
**a** Number of currently completed studies per year of primary study completion and the corresponding number of studies with available results. **b** Cumulative number of patients enrolled into studies per year and the corresponding cumulative number of patients enrolled into studies with available results

The number of completed studies and the respective number of studies with results currently available are shown in Fig. 2a. Very few studies on pediatric dialysis were registered as completed before 2013. Figure 2b shows the cumulative number of enrolled study participants in all studies by year of primary study completion. The cumulative high number of patient enrollment results from one large register study including 1892 patients completed in 2019 (NCT02960867).

### Time from primary completion until public availability of study results

**Fig. 3:**
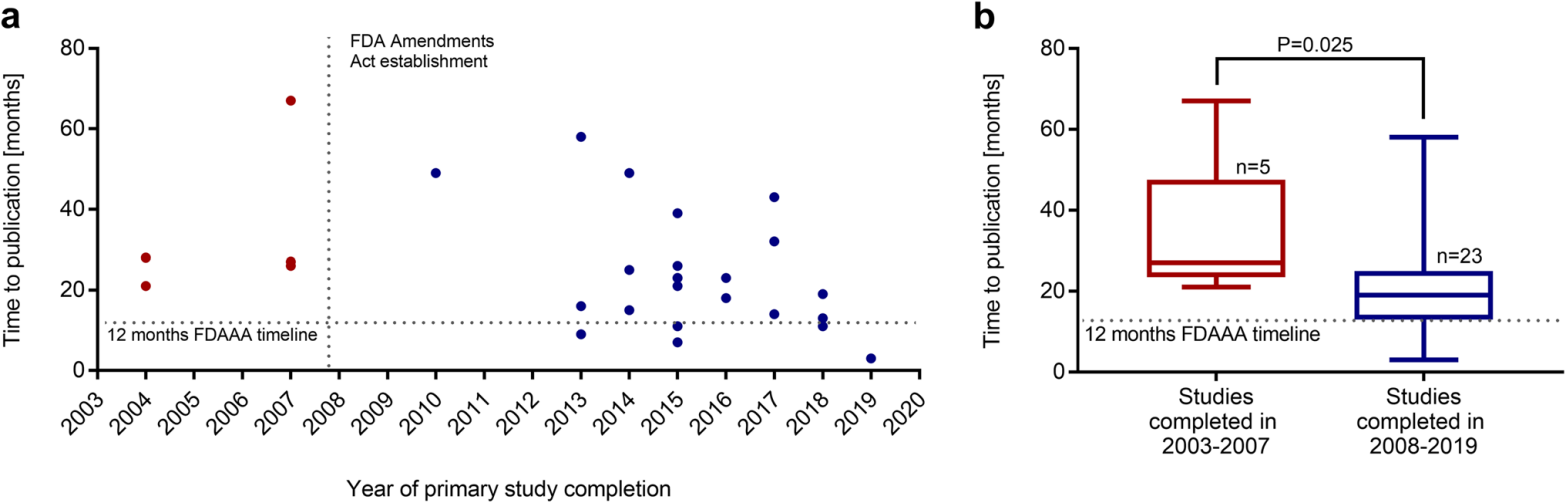
**a** Time from completion to availability of results by year of primary study completion. Each dot represents one completed study. The date of the establishment of the FDA Amendments Act is indicated by the vertical dotted line. The maximal recommended timeframe of 12 months between primary study completion and publication of results as mandated by the FDA is marked by the horizontal dotted line. **b** Median time to publication of completed studies until 2007 and since 2008 after the establishment of the FDA Amendments Act. The maximal recommended timeframe of 12 months between primary study completion and publication of results as mandated by the FDA is marked by the horizontal dotted line. *FDA, Federal Drug Administration*

Figure 3 shows the duration from primary study completion until availability of study results. The median time to public availability of results was 20.5 months (IQR 14–26.8; range 3–67). The median time to peer-reviewed publication was 23 months (IQR 15–32; range 3–60). Figure 3a shows that more studies on pediatric dialysis were published in recent years and that the time to publication was shorter in comparison. Studies completed after 2007, when the FDAAA first became effective, had a significantly (P = 0.025) shorter median time to publication (27 months, IQR 23.5–47.5 vs. 19 months, IQR 13–25) (Fig. 3b). The FDAAA timeline of 12 months from primary completion until publication was only achieved by six studies. All of those had been completed after 2007.

### Factors associated with the publication rate

**Fig. 4:**
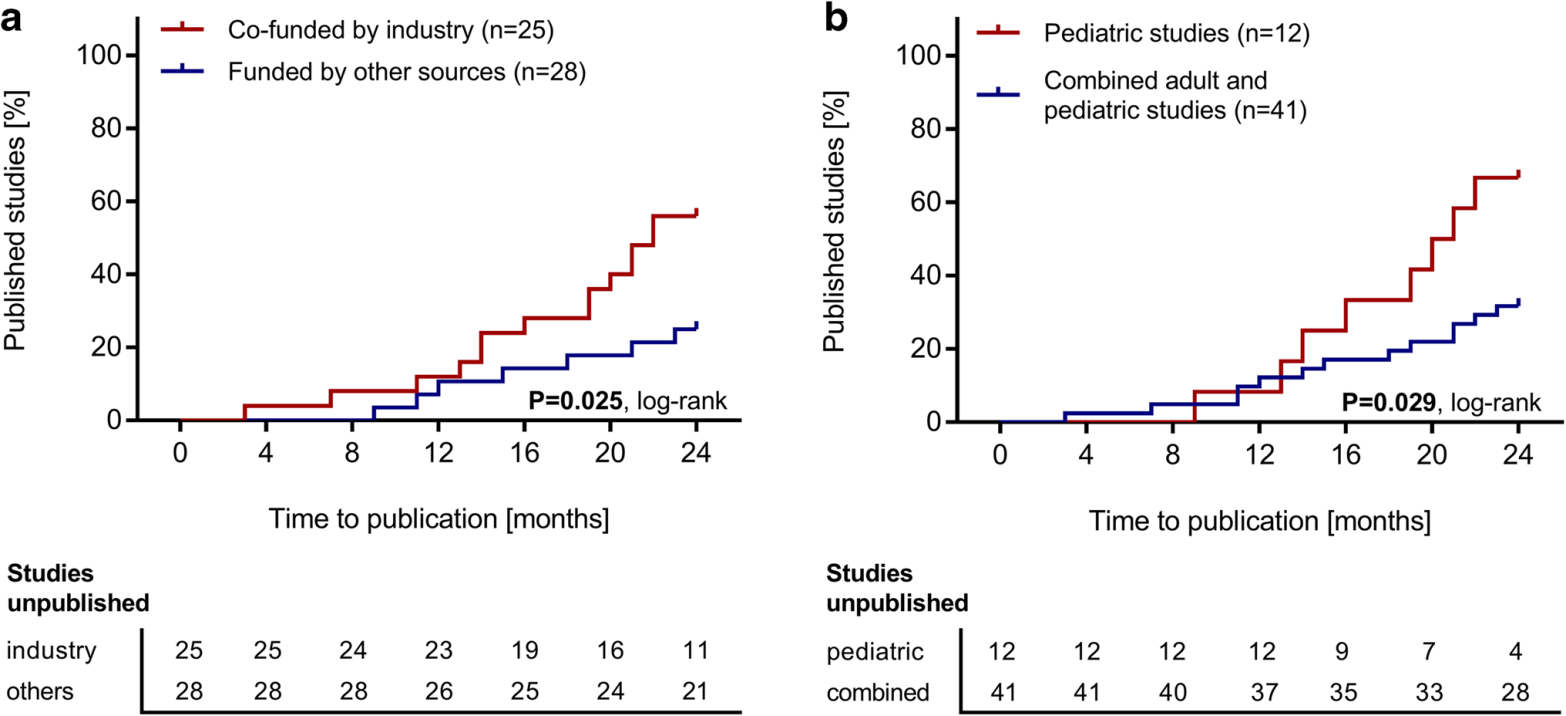
**a** Cumulative percentage of studies on pediatric dialysis registered on ClinicalTrials.gov according to the time from primary study completion until availability of study results within 24 months after primary study completion and stratified according to funding type. **b** Cumulative percentage of studies on pediatric dialysis registered on ClinicalTrials.gov according to the time from study completion until availability of study results within 24 months after primary completion and stratified according to enrollment of children only *vs.* enrollment of both adults and children

**Fig. 5:**
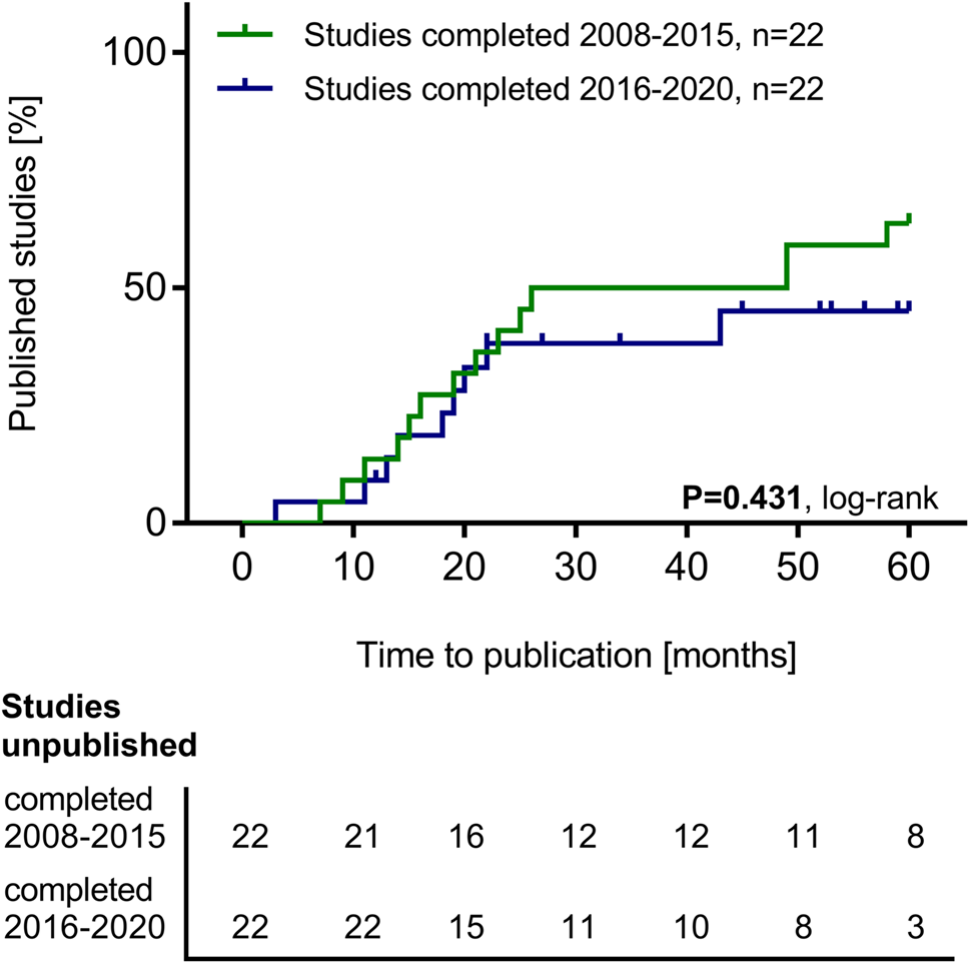
Cumulative percentage of studies on pediatric dialysis registered on ClinicalTrials.gov according to the time from primary study completion until availability of study results within 60 months after primary study completion and stratified according to the year of completion (2008–2015 and 2016–2020)

Studies co-funded by industry had a significantly higher 2-year publication rate (P = 0.025; Fig. 4a). The type of funding had no influence on the 5-year publication rate (Supplementary Fig. 1a). Pediatric studies had both a significantly higher 2-year and 5-year publication rate (P = 0.029 and P = 0.038; Fig. 4b and Supplementary Fig. 1b). Research topics did not significantly impact the 2-year and 5-year publication rate and there was also no significant difference in the 2-year and 5-year publication rates between RCTs and non-randomized controlled trials (Table 3). The median impact factor value was neither associated with early (published within 2 years after primary completion) nor with late publication (published after 2 years, but not later than 5 years post primary completion) (Table 2). A comparison of studies completed between 2008 after the FDAAA until 2015 and studies completed between 2016 and 2020 revealed no significant difference regarding the 5-year publication rate (Fig. 5).

**Table 3:** Factors associated with the 2-year and 5-year publication rate in completed pediatric dialysis studies

Factors associated with the publication rates	2-year publication rate (Hazard ratio, 95% CI)	P value (log rank)	5-year publication rate (Hazard ratio, 95% CI)	P value (log rank)
Focus on medication and control of secondary complications vs. other focus	42.3% vs. 37.0% (1.08, 0.46–2.52)	0.843	52.9% vs. 56.4% (0.92, 0.44–1.97)	0.838
Focus on technical aspects and dialysis prescription vs. other focus	30.0% vs. 45.5% (0.58, 0.25–1.39)	0.257	53.6% vs. 55.3% (0.84, 0.39–1.81)	0.670
Co-funding by industry vs. other funding sources	56.0% vs. 25.0% (2.66, 1.13–6.33)	0.025	63.5% vs. 46.2 (1.77, 0.83–3.83)	0.127
Pediatric studies vs. studies enrolling both children and adult patients	66.7% vs. 31.7% (2.54, 0.86–7.51)	0.029	75.0% vs. 48.2% (2.25, 0.85–6.00)	0.038
Randomized controlled studies vs. non-randomized controlled studies	21.4% vs. 46.2% (0.38, 0.15–0.99)	0.111	40.0% vs. 47.7% (0.95, 0.42–2.17)	0.918

Abbreviations: *CI*, confidence interval

### Analysis of most recently completed studies

Twelve studies had a primary completion date within 5 years until the close of database (November 20, 2017 until November 20, 2021). Four of these 12 studies (33%) had results available within this time frame (3 peer-reviewed publications). The median time to public availability of results was 12 months. Three studies had a primary completion date within 2 years until the closure of database (November 20, 2019 until November 20, 2021). None of these studies had results available within this time frame.

## Discussion

The main finding of this analysis on the current publication rate of completed studies in pediatric dialysis is that half of the studies registered on ClinicalTrials.gov with a primary completion date between the years 2003 and 2020 currently remain unpublished. We consider this a significant problem. Children on maintenance dialysis represent a vulnerable patient group with high disease burden and comorbidity as well as a limited quality of life. A resulting publication bias arising from presumable non-submission of statistically insignificant or negative study results in the field of pediatric dialysis might negatively impact clinical decision-making regarding the treatment of these vulnerable children [21–23].

Despite regulatory measures taken against non-submission of study results, for example the FDAAA from 2007 (which became final US law in 2017, “Final Rule”), or the ICMJE statement from 2004 and the AllTrials campaign, the reported publication rate of clinical studies in general is currently neither complete nor are the results published in a timely fashion. This was shown for different pediatric subspecialties and in adult medicine by several, partly high-ranking, publications [9, 10, 17, 18, 24, 25]. Our results show that an incomplete publication rate is also found in the specialized field of pediatric dialysis. Whereas recent pediatric analyses had reported the lowest percentage of publications in pediatric liver transplantation of only 58% until the respective close of database, we could show that the publication rate in pediatric dialysis is currently even lower [10].

The FDAAA does not only require the publication of all study results but also requires these results to be published within 12 months after primary study completion [18]. In our analysis on pediatric dialysis studies, we showed that respective studies were published faster since the first establishment of the FDAAA in 2007. However, only a small fraction of these studies had results available within 12 months after their primary completion and the median time to publication for studies completed after 2007 was still nearly 2 years. Similar findings were shown in adult and pediatric medicine recently. These also demonstrate an unacceptably long time to publication, but regulatory measures like the FDAAA were shown to inflict improvement [9, 10, 18]. Yet, even if the time to publication of pediatric dialysis studies improved after the FDAAA establishment, we could show no significant improvement regarding the overall publication rate in pediatric dialysis studies over time. Studies completed in the first 6 years after the FDAAA had a similar 5-year publication rate as compared to studies completed thereafter.

The reasons for non-submission or late submission of study results in medicine are not always known; however, some potentially influencing factors like the source of funding have been shown to be associated with the publication rate [18]. We also examined potential influencers on the publication rate and the time to publication in our study cohort. We found that pediatric dialysis studies did not seem to be preferably funded by industry. Half of all identified studies were funded by academia. Other researchers report higher rates of co-funding by industry in other areas of medicine [18]. However, pediatric dialysis studies co-funded by industry had a higher publication rate within 2 years, but not within 5 years, after completion than studies without an industrial sponsor. Other than academia, industrial sponsors usually can provide more resources to promote faster processing and analysis of study results which might explain these findings. Anderson et al. recently reported similar results in an analysis on the publication rate in adult medicine [18, 26]. One-fourth of all investigated studies had a randomized controlled design. However, this was not associated with a higher or lower publication rate within 2 or 5 years after completion. So, in the field of pediatric dialysis, the complexity of the studies does not necessarily seem to influence the publication rate. However, we observed that two-thirds of studies with currently available results and more than 80% of studies with peer-reviewed publications had an interventional design. This result indicates that among the currently published studies in pediatric dialysis, those studies with better evidence-grading clearly predominate, which is reassuring. Further, purely pediatric studies, which are likely more relevant and applicable to the field than studies enrolling both adult and pediatric patients, were also published more often (75%).

We further identified two major research topics in pediatric dialysis studies, (i) medication during dialysis and/or control of secondary complications of kidney failure and (ii) dialysis technique and efficiency. Nearly half of all studies on pediatric dialysis focused on medication and control of secondary complications, indicating a persistent need for further improvement in therapeutic chronic kidney disease management. We further found that studies which only focused on children were published faster and had a higher 5-year publication rate, compared to those studies which enrolled both children and adults. We could also show that studies which only focused on children had a significantly smaller cumulative enrollment, which might explain the faster publication rate. Lower patient numbers may limit the choice of statistical methods that can be reasonably performed, thereby require fewer resources and thus lead to a faster overall data analysis.

Our study has several limitations. First, we did not consult databases for trial registration other than ClinicalTrials.gov. ClinicalTrials.gov is currently the largest, most widely used and appreciated database for the registration of clinical studies, and it assesses study details completely as required by the FDA [20, 24]. In principle, the reference points for publication of results and time to publication as mandated by the FDAAA can only be applied to trials that are registered on ClinicalTrials.gov. Second, the requirements of the FDAAA in principle apply only to so-called “Applicable Trials” under the FDAAA [20]. ClinicalTrials.gov did not provide information about which studies were applicable and which were not. However, we believe that all clinical studies should be published in a timely manner after completion. Therefore, we measured the time to publication against the 12-month timeline as mandated by the FDAAA for all studies in our analysis. Third, we only included studies which were completed until November 20, 2020 and ended our search for publications on November 20, 2021 in order to consider all available publications within 12 months from primary study completion. However, most studies in our analysis had been published beyond the 12-month timeline mandated in the FDAAA. Thus, our analysis of the current percentage of studies with available results and of time to publication might miss results of more recently completed studies, which might still become available after database closure (Fig. 2a/b, Fig. 3b and Table 1). We tried to overcome this potential source of bias by analyzing the 5-year-publication rates based on the Kaplan–Meier method (Supplementary Fig. 1 and Fig. 5). Fourth, an analysis performed on studies with a primary completion date within 5 years and within 2 years until the closure of database on the one hand confirmed the trend toward more timely publication in more recent years. On the other hand, the publication rate in this analysis was comparably low (0–30%). We can only speculate whether this rate might improve within the next years after the closure of our database. Fifth, we had no information about possible legally acceptable exceptions, as stated in the FDAAA, regarding a delay of study results publication for studies sponsored by industry [18]. Sixth, pediatric dialysis is a highly specific subspecialty of pediatric nephrology and study results from adult dialysis medicine must not be extrapolated uncritically to children. However, it would have added to the information content of this study if the publication rate in pediatric dialysis had been compared with that from adult medicine. We found 915 completed adult dialysis studies on ClinicalTrials.gov at the time of database closure; however, an analysis of the publication rate of these studies was beyond our resources and the scope of this study.

## Conclusion

Our analysis of the current publication rate in completed studies on pediatric dialysis revealed a low overall number of publications. Only half of all studies had currently available study results, and there was no significant improvement of the publication rate over time. Besides that, the time from primary completion to publication did not meet FDAAA requirement levels, even if an improvement with respect to faster publications became evident in recent years. It is likely that a publication bias results from the lack of published results, which could negatively impact clinical decision-making in pediatric dialysis. We were able to identify some relevant factors influencing the publication rate in pediatric dialysis, yet most of the assumable reasons for non-publication or late publication in the field remain purely speculative and thus unknown. Furthermore, we consider the high number of patients in currently unpublished pediatric dialysis studies problematic from an ethical point of view. The low publication rates entail a certain risk that accomplished but unpublished studies in the long run be repeated.

## Supplementary Information

Below is the link to the electronic supplementary material.

Supplementary file1 (DOCX 152 kb)

Graphical Abstract (PPTX 264 kb)

## Data Availability

All data generated or analyzed during this study are included in this published article and its online supplemental files. The respective clinical studies analyzed in this article can be accessed via ClinicalTrials.gov. Search criteria are given in the methods section of this manuscript.

## Abbreviations

CI: Confidence interval
FDAAA: FDA Amendments Act
ICMJE: International Committee of Medical Journal Editors
RCT: Randomized controlled trial
USRDS: United States Renal Data System
