# Supplementary material for: Missing trial results: analysis of the current publication rate of studies in pediatric dialysis from 2003 to 2020
Source: Pediatr Nephrol. 2022 Apr 23;38(1):227–36. doi: 10.1007/s00467-022-05553-x (PMC9747852; doi:10.1007/s00467-022-05553-x)
Supplement: Supplementary file 1 — Supplementary file1 (DOCX 152 kb) [file 467_2022_5553_MOESM1_ESM.docx]

**Supplemental Material**

**Supplemental Figure 1**


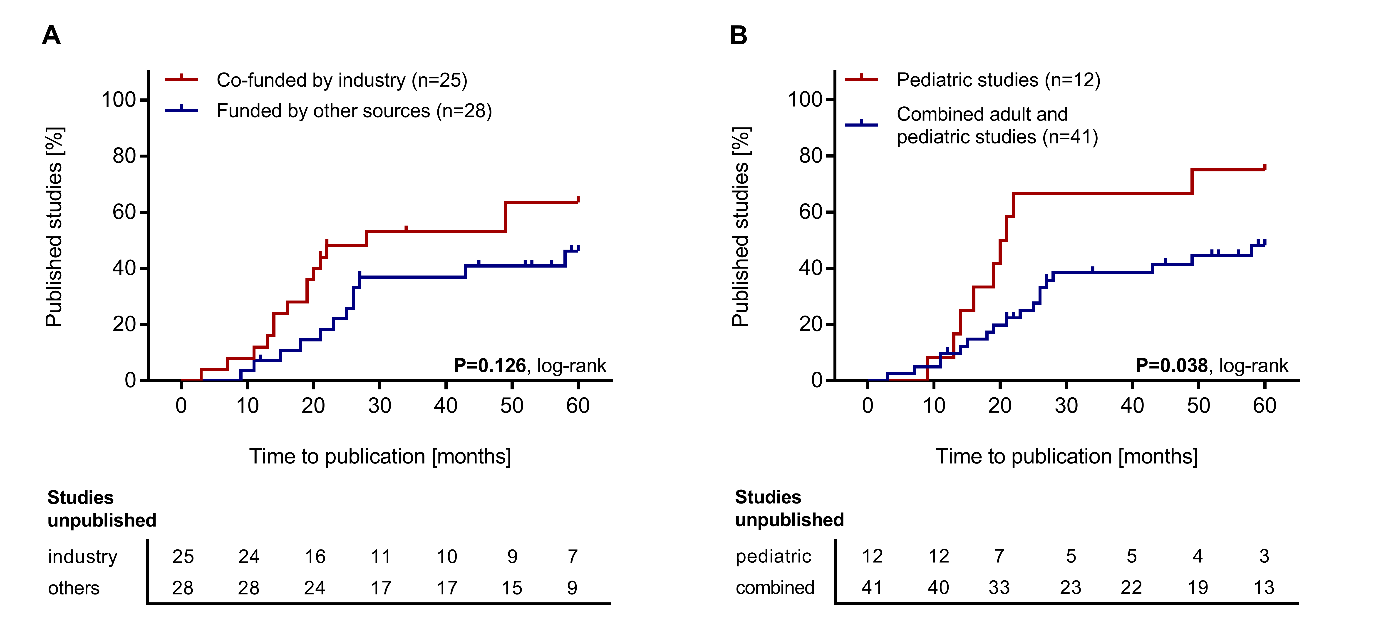


**Supplemental Figure 1. (a)** Cumulative percentage of studies on pediatric dialysis registered on ClinicalTrials.gov by time from primary study completion till availability of study results within 60 months after completion and stratified according to funding type **(b)** Cumulative percentage of studies on pediatric dialysis registered on ClinicalTrials.gov by time from primary study completion till availability of study results within 60 months after completion and stratified according to enrollment of children only and enrollment of both adults and children.

**Supplemental Table 1: Studies in pediatric dialysis with focus on medication and control of secondary complications of kidney failure.** Studies enrolling only pediatric patients are marked as “pediatric”. Studies are sorted by date of primary study completion.

| **Nr.** | **Study title on ClinicalTrials.gov** | **Year of primary completion** | **NCT number** | **Study design** | **Phase** | **Funded by industry** | **Pediatric** | **Available results** |
| --- | --- | --- | --- | --- | --- | --- | --- | --- |
| 1 | Study of the Efficacy of Two Doses of Ferrlecit in the Treatment of Iron Deficiency in Pediatric Hemodialysis Patients | 2003 | NCT00223964 | RCT | 4 | yes | yes | no |
| 2 | Pharmacokinetic Study of Intravenous Iron Sucrose in Adolescents on Hemodialysis or Peritoneal Dialysis Receiving Epoetin | 2003 | NCT00239616 | non-RCT interventional | 4 | yes | no | no |
| 3 | NESP Pediatric Study | 2004 | NCT00527137 | RCT | 3 | yes | no | yes |
| 4 | A Long Term Study of Lanthanum Carbonate in Patients Requiring Dialysis Who Have Ived Lanthanum Carbonate in Previous Studies Defined by the Protocol. | 2006 | NCT00150540 | non-RCT interventional | 3 | yes | no | no |
| 5 | Glycyrrhetinic Acid-Effect on Serum Potassium and Insulin Resistance in Dialysis Patients | 2007 | NCT00384384 | RCT | 2 | no | no | yes |
| 6 | Audit of the Effect of Changing From IV to SC Administration of ESA's in Haemodialysis Patients | 2007 | NCT00382044 | observational |  | no | no | no |
| 7 | A Study of MCI-196 in Chronic Kidney Disease Stage V Subjects on Dialysis With Hyperphosphatemia | 2010 | NCT00542815 | non-RCT interventional | 3 | yes | no | yes |
| 8 | Study of the Influence of Dosages of 1-84 and 7-84 Fragments of Parathyroid Hormone Compared to Conventional Dosage on the Balance of Calcium and Phosphate in Hemodialysis (PTH) | 2011 | NCT02859220 | RCT | NA | no | no | no |
| 9 | Three Times Weekly (TIW) Growth Hormone Therapy in Children on Hemodialysis | 2012 | NCT00943995 | RCT | 3 | yes | yes | no |
| 10 | European Union Registry in Paediatric Chronic Kidney Disease Patients Looking at Safety and Usage Patterns of Darbepoetin Alfa | 2013 | NCT00838097 | observational | NA | yes | yes | yes |
| 11 | Fosrenol Post-marketing Surveillance for Continuous Cyclic Peritoneal Dialysis in Japan | 2014 | NCT01412398 | observational | NA | yes | no | no |
| 12 | Study of the Pharmacokinetics of Daptomycin in Children With Renal Disease | 2014 | NCT01012089 | non-RCT interventional | NA | yes | yes | yes |
| 13 | A Study to Evaluate the Safety of Paricalcitol Capsules in Pediatric Subjects Ages 10 to 16 With Stage 5 Chronic Kidney Disease Receiving Peritoneal Dialysis or Hemodialysis | 2015 | NCT01382212 | non-RCT interventional | 3 | yes | yes | yes |
| 14 | Antibody Response to Human Papillomavirus Recombinant Vaccine (Gardasil®) in Girls and Young Women With Chronic Kidney Disease | 2015 | NCT00806676 | non-RCT interventional | NA | yes | no | yes |
| 15 | Optimal Administration of Allopurinol in Dialysis Patients | 2015 | NCT02477488 | non-RCT interventional | 4 | no | no | no |
| 16 | Immunogenicity of Fluzone High Dose in Immunocompromised Children and Young Adults | 2015 | NCT01685372 | RCT | 2 | no | no | yes |
| 17 | Study to Evaluate Cinacalcet in Children With Chronic Kidney Disease | 2015 | NCT01290029 | non-RCT interventional | 1 | yes | yes | yes |
| 18 | Fosrenol Post-marketing Surveillance in Japan | 2016 | NCT01955876 | observational | NA | yes | no | no |
| 19 | Triferic Pediatric Pharmacokinetic Protocol | 2016 | NCT02595437 | non-RCT interventional | 1-2 | yes | yes | yes |
| 20 | Extension Study of Cinacalcet for Treatment of Secondary Hyperparathyroidism (SHPT) in Pediatric Patients With Chronic Kidney Disease on Dialysis | 2017 | NCT02341417 | non-RCT interventional | 3 | yes | no | yes |
| 21 | A Single-dose Study in Paediatric Patients Aged 2 to Less Than 18 Years With Secondary Hyperparathyroidism (sHPT) Receiving Haemodialysis (and Etelcalcetide) | 2017 | NCT02833857 | non-RCT interventional | 1 | yes | yes | yes |
| 22 | A Study to Assess the Pharmacokinetics of Lanthanum Carbonate, Investigate and Compare the Efficacy, Safety and Tolerability of Lanthanum Carbonate With Calcium Carbonate in Hyperphosphataemic Children and Adolescents With Chronic Kidney Disease on Dialysis | 2018 | NCT01696279 | non-RCT interventional | 2 | yes | yes | yes |
| 23 | Post-marketing Surveillance of Bixalomer in Patients With Pre-dialysis Chronic Kidney Disease | 2018 | NCT02805348 | observational | NA | yes | no | no |
| 24 | Carnitine, Aclycarnitine, Myocardial Function, and CRRT | 2019 | NCT01941823 | observational | NA | no | no | no |
| 25 | Post-Marketing Surveillance on Long-Term Drug Use of Kiklin (Bixalomer)® Capsules in Patients With Hyperphosphatemia Receiving Peritoneal Dialysis | 2019 | NCT01903213 | observational | NA | yes | no | no |
| 26 | Study to Evaluate the Efficacy and Safety of Oxabact (OC5) in Primary Hyperoxaluria Patients Who Are on Dialysis | 2020 | NCT02000219 | non-RCT interventional | 2 | yes | no | no |

*RCT, randomized controlled trial; NESP, novel erythropoietin stimulating protein (darbepoetin alpha)*

**Supplemental Table 2: Studies in pediatric dialysis with focus on dialysis technique and efficiency.** Studies enrolling only pediatric patients are marked as “pediatric”. Studies are sorted by date of primary study completion.

| **Nr.** | **Study title on ClinicalTrials.gov** | **Date of primary completion** | **NCT number** | **Study design** | **Phase** | **Funded by industry** | **Pediatric** | **Available results** |
| --- | --- | --- | --- | --- | --- | --- | --- | --- |
| 1 | Open Label Trial to Establish the Equivalence Between ANDY-Disc® and Ultrabag® in Patients on CAPD. | 2004 | NCT00255619 | non-RCT interventional | 4 | yes | no | yes |
| 2 | Administration of pH-Neutral Peritoneal Dialysis Solutions Containing Lactate or Bicarbonate in Children | 2007 | NCT01632046 | RCT | 4 | no | no | yes |
| 3 | Evaluation of a Simplified Protocol for Regional Citrate Anticoagulation in Continuous Venovenous Hemodiafiltration | 2008 | NCT00583765 | observational | NA | yes | no | no |
| 4 | More Frequent Dialysis (>3 Treatments Per Week) | 2012 | NCT00575497 | non-RCT interventional | NA | no | no | no |
| 5 | More Frequent In-Center Hemodialysis in Pediatric End Stage Renal Disease | 2013 | NCT01352455 | RCT | NA | no | no | yes |
| 6 | Prophylactic Peritoneal Dialysis Decreases Time to Achieve a Negative Fluid Balance After the Norwood Procedure | 2013 | NCT01215240 | RCT | NA | no | yes | yes |
| 7 | Observational Study of Citrate Based Dialysis in Pediatric Patients Receiving Hemodialysis | 2014 | NCT01590550 | observational | NA | no | no | yes |
| 8 | Effect of Varied Dialysate Bicarbonate Levels on Phosphate and Potassium Removal | 2014 | NCT01930370 | non-RCT interventional | 4 | no | yes | no |
| 9 | Peritoneal Dialysis vs Furosemide for Acute Kidney Injury After Cardiopulmonary Bypass | 2015 | NCT01709227 | RCT | NA | no | yes | yes |
| 10 | Post Marketing Surveillance of the CentrosFLO™ Tunneled IJ Catheter | 2015 | NCT02349308 | observational | NA | yes | no | yes |
| 11 | Hemodialysis Blood Flow and Urea Clearance | 2015 | NCT02484118 | non-RCT interventional | NA | no | no | yes |
| 12 | Role of CVVH in Patients With Acute Paraquat Poisoning | 2016 | NCT01709604 | observational | NA | no | no | yes |
| 13 | Estimating and Predicting Hemodynamic Changes During Hemodialysis | 2016 | NCT01700465 | observational | NA | no | no | no |
| 14 | Magnesium Balance of Citrate-based Continuous Venovenous Hemofiltration, Effect of Citrate Dose. | 2017 | NCT02194569 | RCT | NA | no | no | no |
| 15 | Prophylactic Omentopexy During Laparoscopic Insertion of Peritoneal Dialysis Catheter | 2017 | NCT02879734 | RCT | NA | no | no | no |
| 16 | DiaSport - Endurance-orientated Training Program With Children and Adolescents on Maintenance Hemodialysis | 2017 | NCT01561118 | RCT | NA | no | no | yes |
| 17 | Association of Survival in Impact of Ultrafiltration Rates in Hemodialysis Patients | 2018 | NCT03471299 | observational | NA | no | no | no |
| 18 | Human Factors Validation Testing for the Peripal System; a Manual Connection Assist Device for Peritoneal Dialysis Devices | 2018 | NCT04292210 | non-RCT interventional | NA | yes | no | yes |
| 19 | Uraemic Toxins in Chronic Kidney Disease Paediatric Patients: Observational Study | 2019 | NCT02624466 | observational | NA | no | no | no |
| 20 | Outcomes of Renal Access Arteriovenous Fistulas for Hemodialysis in Patients With Chronic Renal Failure | 2019 | NCT04386954 | observational | NA | no | no | no |

*RCT, randomized controlled trial*

**Supplemental Table 3: Studies in pediatric dialysis with focus on other topics.** Studies enrolling only pediatric patients are marked as “pediatric”. Studies are sorted by date of primary study completion.

| **Nr.** | **Study title on ClinicalTrials.gov** | **Date of primary completion** | **NCT number** | **Study design** | **Phase** | **Funded by industry** | **Pediatric** | **Results**  **available** |
| --- | --- | --- | --- | --- | --- | --- | --- | --- |
| 1 | Biotin Deficiency and Restless Legs Syndrome | 2007 | NCT02011191 | RCT | NA | no | no | yes |
| 2 | Study of Patients With Acute Renal Failure on CVVH | 2010 | NCT00207909 | observational | NA | no | no | no |
| 3 | Sleep Disorders in Pediatric Dialysis | 2014 | NCT01667588 | observational | NA | no | no | yes |
| 4 | Association Between Depression and Iron Metabolism in Hemodialysis Patients | 2017 | NCT03096626 | observational | NA | no | no | no |
| 5 | Association of Mean Platelet Volume and Cardiovascular Disease in Children With End Stage Renal Disease. | 2018 | NCT03759002 | observational | NA | no | no | yes |
| 6 | European Registry of Dialysis Treatment of Pediatric Acute Kidney Injury (AKI) | 2019 | NCT02960867 | observational | NA | yes | no | yes |
| 7 | Understand the Association Between Peritoneal Dialysis Status and Gene Polymorphisms of VEGF and KDR | 2020 | NCT04888065 | observational | NA | no | no | no |

*RCT, randomized controlled trial*
