# Supplementary material for: Missing trial results: analysis of the current publication rate of studies in pediatric dialysis from 2003 to 2020
Source: Pediatr Nephrol. 2022 Apr 23;38(1):227–36. doi: 10.1007/s00467-022-05553-x (PMC9747852; doi:10.1007/s00467-022-05553-x)
Supplement: Supplementary file 2 — Graphical Abstract (PPTX 264 kb) [file 467_2022_5553_MOESM2_ESM.pptx]

## Slide 1
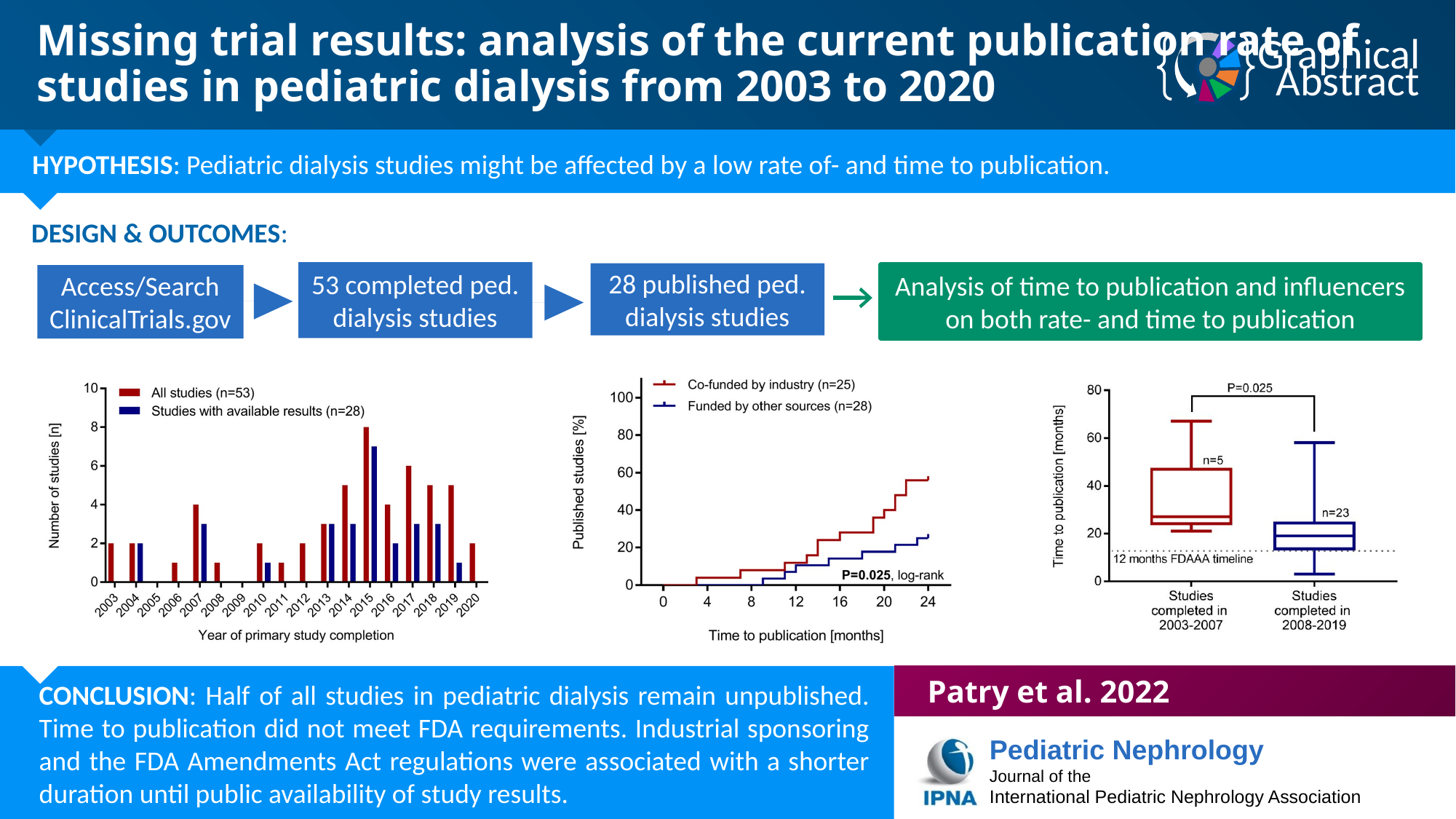

Missing trial results: analysis of the current publication rate of
studies in pediatric dialysis from 2003 to 2020
HYPOTHESIS: Pediatric dialysis studies might be affected by a low rate of- and time to publication.
DESIGN & OUTCOMES:
53 completed ped. dialysis studies
28 published ped. dialysis studies
Analysis of time to publication and influencers on both rate- and time to publication
Access/Search
ClinicalTrials.gov
Patry et al. 2022
CONCLUSION: Half of all studies in pediatric dialysis remain unpublished. Time to publication did not meet FDA requirements. Industrial sponsoring and the FDA Amendments Act regulations were associated with a shorter duration until public availability of study results.
